# Assessing the effects of climate change on arthropod abundance in Azorean pastures: PASTURCLIM project's baseline monitoring data

**DOI:** 10.3897/BDJ.11.e103723

**Published:** 2023-04-28

**Authors:** Sophie Wallon, Catarina Melo, Noelline Tsafack, Rui B. Elias, Paulo A.V. Borges

**Affiliations:** 1 Centre for Ecology, Evolution and Environmental Changes (cE3c)/Azorean Biodiversity Group, CHANGE – Global Change and Sustainability Institute, Faculty of Agricultural Sciences and Environment, University of the Azores, Rua Capitão João d´Ávila, Pico da Urze, 9700-042, Angra do Heroísmo, Azores, Portugal Centre for Ecology, Evolution and Environmental Changes (cE3c)/Azorean Biodiversity Group, CHANGE – Global Change and Sustainability Institute, Faculty of Agricultural Sciences and Environment, University of the Azores, Rua Capitão João d´Ávila, Pico da Urze, 9700-042 Angra do Heroísmo, Azores Portugal; 2 CFE – Centre for Functional Ecology, 3001-401 Coimbra, Portugal CFE – Centre for Functional Ecology 3001-401 Coimbra Portugal; 3 Regional Secretariat of Environment and Climate Change, Project LIFE BEETLES (LIFE 18NAT/PT/000864), Rua do Galo n118, 9700-040, Angra do Heroísmo, Azores, Portugal Regional Secretariat of Environment and Climate Change, Project LIFE BEETLES (LIFE 18NAT/PT/000864), Rua do Galo n118, 9700-040 Angra do Heroísmo, Azores Portugal; 4 IUCN SSC Mid-Atlantic Islands Invertebrate Specialist Group, Angra do Heroísmo, Azores, Portugal IUCN SSC Mid-Atlantic Islands Invertebrate Specialist Group Angra do Heroísmo, Azores Portugal

**Keywords:** arthropods, climate change, grasses, Open Top Chamber, pasture, pitfall traps.

## Abstract

**Background:**

The data we present are part of the project PASTURCLIM (Impact of climate change on pasture’s productivity and nutritional composition in the Azores). The project aims to assess the consequences of climate change (e.g. temperature increase) on the grass production and its quality for forage, as well as to assess changes in the arthropod communities associated with the Azorean intensive pastures. An *in situ* experiment was set up using Open Top Chambers (OTCs), in order to simulate an increasing of temperature (average of +1.2ºC) on pastures. In this contribution, we present the data relative to the arthropod sampling.

**New information:**

We provide an inventory of all arthropods recorded inside OTCs and in control plots in three intensively managed pastures dominated by grasses in Terceira Island (Azores): two of them dominated by ryegrass, *Loliummultiflorum* Lam. (Poaceae), located respectively at 186 m and 301 m above sea level; and one field dominated by common velvetgrass, *Holcuslanatus* L. (Poaceae), located at an altitude of 385 m.

A total of 41351 specimens were collected. Organisms collected belong to four classes, 15 orders, 60 families and 171 species/morphospecies (including 34 taxa identified only at order, family or genus level). Therefore, for only 137 taxa, we have a scientific name associated (n = 38918). A total of 75% of the species (n = 129 species) are considered introduced (including all the species with indeterminate colonisation status that are possibly also exotic species (n = 7622)), representing 71% of the total abundance (n = 29664 specimens). A total of 19% of the species (n = 33 species) are considered native non-endemic representing 28% of the total abundance (n = 11608 specimens). Only one endemic species was sampled, the wolf spider *Pardosaacorensis* Simon, 1883 (1% of the species), representing 0.2% of the total abundance (n = 79 specimens). Spiders (5056 specimens) and beetles (18310 specimens) were the dominant taxa representing, respectively, 20 and 78 morphospecies.

Since the main aim of this study was to have a better knowledge on arthropod communities present in Azorean pastures under a simulated temperature increase, the principal novelty of this paper is the contribution with distribution and abundance data to a baseline knowledge on the future consequences of climate changes on arthropod communities in Azorean pastures.

## Introduction

Climatic changes occurring on Earth imply mainly changes in temperature (Arnell et al. 2019, Pörtner et al. 2022) and in rainfall patterns (Ohba and Sugimoto 2019, Papalexiou and Montanari 2019), which affect ecosystems as well as their biodiversity (Sharma and Dhillon 2018, Habibullah et al. 2022). Grasslands used as forage crops are affected at different levels by the increase of temperature: i) Increased growth rate, in which higher temperatures can stimulate the growth rate of forage crops. As a result, grasslands can produce more forage, which can be beneficial for livestock. However, changes in seasonal precipitation would reduce these benefits, particularly in areas with low summer rainfall (Hopkins and Del Prado 2007); ii) Drought stress, in which higher temperatures comes with the higher risk of drought, which can be detrimental to the growth of forage crops. Drought stress can reduce the yield of grasslands and result in poor-quality forage; iii) Changes in plant composition (Feeley et al. 2020), in which some species may become less abundant, while others may thrive, which can alter the nutritional value of the forage, decreasing protein and mineral nutrient concentrations, as well as altering lipid composition (DaMatta et al. 2010); iv) Changes in plant phenology, in which some grasses are affected as well as their functional traits and chemical composition (Lee et al. 2013, Piao et al. 2019, Ekholm et al. 2020, Melo et al. 2022). All these factors can lead to cascading effects on biodiversity and on ecosystem services (Selvaraj et al. 2013, Banerjee et al. 2018, Garcia et al. 2018, Mirás-Avalos and Baveye 2018, Moss and Evans 2022).

Adaptation to climate change for agriculture will be definitively a crucial point to overpass in order to avoid an economic crisis in the coming years (Aguiar et al. 2018, Rivera et al. 2018, Vizinho et al. 2021). Regarding wildlife, there has been great concern for many years concerning the decline of arthropods (Wilson 1987, Brantley and Ford 2012, Seibold et al. 2019, Halsch et al. 2021). In anthropised ecosystems such as for crops or pastures, they are responsible for many ecosystem services (e.g. pollination, decomposition of organic matter, pest control and predation), but can also be responsible for ecosystem disservices (e.g. pest, parasitism, herbivory, seed predation, crop damage) (Cardenas et al. 2022, Ferrante et al. 2022). Climate changes may also affect this balance of services and disservices by inducing shifts in species composition (Harter et al. 2015). Climate changes may influence species presence/absence, fluctuation of abundances and can even favour the dominance of some species in the ecosystem with the threat of creating a boom of pest species (Buchholz et al. 2013, Sohlström et al. 2022). The risk is higher on island ecosystems because of the limited area available and the usually lower altitudinal range. Therefore, climate changes represent a real threat for island biodiversity (Harter et al. 2015, Borges et al. 2019, Veron et al. 2019, Pörtner et al. 2022).

Predictions for the Azores suggest a temperature increase between 1.6 and 2.72°C till the end of the century (respectively following the two scenarios from the PRAC: RCP4.5 and RCP 8.5). Changes in the rainfall pattern are also expected due to the increase in heavy rains and storms in the winter and prolonged droughts during the summer (Costa et al. 2017).

Nowadays, the main activity in the Azores is dairy and meat production. Thus, most of the land between the sea level and middle altitude (500 m) is used for agriculture (e.g. intensive pasture and forage crops) representing 56% of the territory (Costa et al. 2017). The impact of temperature increase on the arthropod communities of Azorean pastures is unknown.

Therefore, an *in-situ* experiment was established to collect baseline data in order to help understand how the increase of the temperature affects the arthropod communities associated with intensive pastures in the Azores.

## General description

### Purpose

To provide baseline data on arthropod species richness and abundance from intensively managed pasture in Terceira Island (Azores) under natural and modified climatic conditions (e.g. increase in temperature via Open Top Chambers - OTCs). These data will allow us to assess the effects of climate change on arthropod’s communities in Azorean pastures.

### Additional information

Open Top Chambers (OTCs) are raised from the floor (around 5 cm) and allow free movement of all crawling arthropods around the pasture. Instead, for flying arthropods, OTCs represent an artificial barrier and data collected would present a bias due to this obstacle. Therefore, we focused on the collection of crawling arthropods using pitfall traps filled with ethylene glycol.

## Project description

### Title

PASTURCLIM - Impact of climate change on pasture’s productivity and nutritional composition in the Azores

### Personnel

Project leaders: Rui B. Elias

Team members: Paulo A. V. B., Sophie Wallon, Catarina D. Melo.

External Consultants : Teresa M. Ferreira.

Parataxonomists: Sophie Wallon; Mauro Matos.

Taxonomist: Paulo A. V. B.

Darwin Core Database management: Paulo A. V. B., Sophie Wallon.

Fieldwork: Sophie Wallon, Catarina D. Melo, Rui B. Elias.

### Study area description

The study was conducted on the Archipelago of the Azores (North Atlantic), on Terceira Island (decimal coordinates 38.712925, -27.234912) which is the third largest island of the Archipelago with 400.2 km^2^ and a maximum altitude above sea level of 1021 m. The Azores are from volcanic origin and have a temperate oceanic climate, relatively wet with mild temperature at low altitude, all year long.

### Design description

The study areas were intensive pastures located at different elevations (Table 1). All pastures were dominated by grasses. The two fields at lower elevations (A and B) were covered by the annual ryegrass, *Loliummultiflorum* Lam. (Poaceae) and the field at higher elevation (C) was covered by the perennial common velvetgrass, *Holcuslanatus* L. (Poaceae).

### Funding

Core funding was obtained from the Project PASTURCLIM (ACORES-01-0145-FEDER-000082) financed by FEDER at 85% and by Azorean Public funds at 15% through the Operational Programme Azores 2020.

Additional funding was secured from the projects FCT-UIDB/00329/2020-2024 (Thematic Line 1 – integrated ecological assessment of environmental change on biodiversity) and Azores DRCT Pluriannual Funding (M1.1.A/FUNC.UI&D/010/2021-2024).

SW is currently being funded by the Ph.D. Grant DRCT - M3.1.a/F/018/2020 (2021-2024).

Darwin-Core and GBIF management were funded by the project Portal da Biodiversidade dos Açores (2022-2023) - PO Azores Project - M1.1.A/INFRAEST CIENT/001/2022.

## Sampling methods

### Study extent

**Figure 1.**
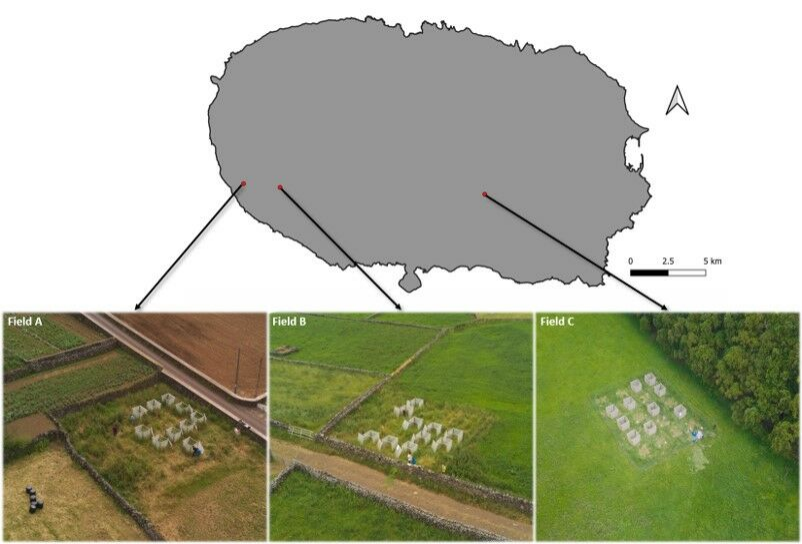
Picture and localisation of each field on the island of Terceira. Each experimental area is covered with 10 control plots and 10 plots surrounded by an OTC (Photo credits: Sebeyes Production).

The study was conducted in three intensive pastures on Terceira Island (Azores) (Fig. 1). In each field, 20 plots (1 x 1 m) were set up in an area of 100m^2^ where cattle were not allowed. Amongst those 20 plots, 10 were randomly chosen to be surrounded by an OTCs (in order to simulate an increase of +1.2ºC average), while the other 10 were considered as control plots. OTCs were built including a 1 m^2^ plot and a margin of 25 cm all around. The aim of this margin was to allow the same set-up of the pitfall traps as in the control plots (e.g. with one pitfall trap at each corner); it also allows free space for scientists to enter inside the OTCs without stepping on the plot. Temperature and relative humidity were recorded through data loggers (Easy Log: EL-USB-2) in control plots and inside OTCs.

### Sampling description

The focus of the study were the arthropods associated with pasture for foraging production. As OTCs represent a physical barrier for flying insects, our focus was made on crawling arthropods. OTCs were raised about 5 cm above the ground and allowed arthropod movement around the experimental area. Pitfall traps were then used for the sampling.

Grasses inside each plot were seasonally and manually harvested to evaluate the biomass. Therefore, pitfall traps were set up and collected before harvesting grasses.

Pitfalls were set up for 14 days, in each field, in the winter of 2020. During the summer of 2020, in the fields A and C, pitfall traps were set up for 14 days, while they were set up for 13 days in Field B.

**Figure 2.**
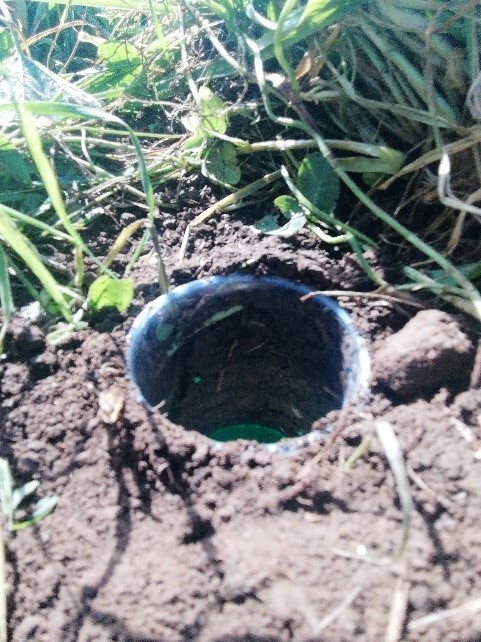
A pitfall trap. The trap was then covered with a plastic dish raised from the ground to avoid overflow of the trap due to eventual rainfall (Photo credit: Sophie Wallon).

Pitfall traps consisted in a 330 ml plastic cups, about 12 cm deep and 8 cm of diameter at the top (Fig. 2). Traps were filled with ethylene glycol. We used car’s cooling liquid at 20% ethylene glycol and added few drops of soap to break the water tension. Specimens collected were then stored into ethanol (96%).

**Figure 3.**
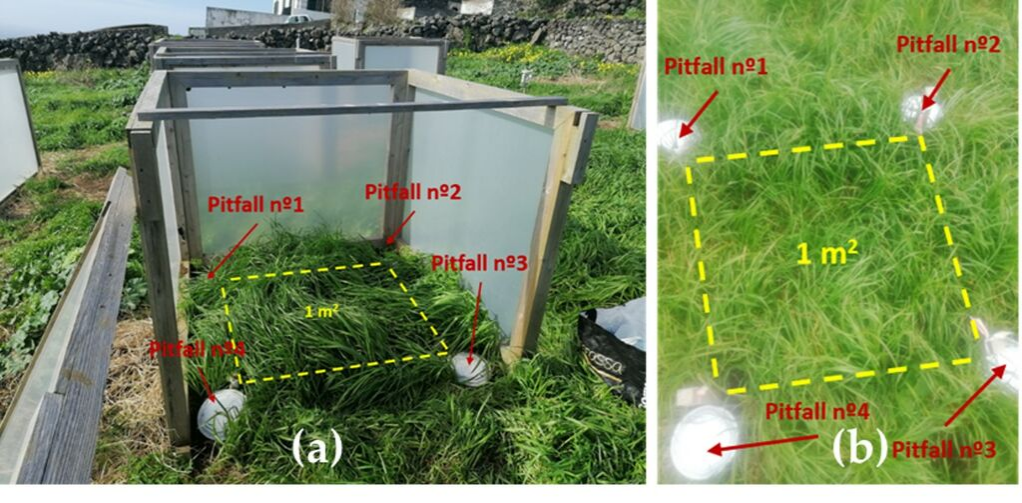
Set-up of an OTC plot (a) and a control plot (b) (Photo credits: Sophie Wallon)

For each season (winter and summer), four pitfall traps were set up on each corner of each plot resulting in four traps per plot (Fig. 3). All traps were were active for 14 days, except during the summer, in field B, where the traps were active for 13 days.

In the winter (March 2020) and before sorting arthropods, the four traps of each plot were merged into one sample corresponding to the plot. For this reason, for the winter 2020 period, only the pitfall number 1 (PTF_1) appears in the column “eventID” that corresponds to four pitfall traps merged into one single sample. Then in the summer (September 2020), each pitfall trap was kept separately before sorting, resulting in four pitfalls for each plot (PTF_1; PTF_2; PTF_3; PTF_4).

In the Event table, the location ID name includes the following information:

Code Site (A, B or C), Control (C) or Treatment with OTCs (T), Plot Number (1 to 10) _ Year of collection - Month of collection_ Pitfall trap (PTF)_ Pitfall number (1 to 4).

For example, the location ID “AC7_2020-09_PTF_3” corresponds to the “Field A Control Plot number 7_ collected in September 2020_ Pitfall trap _ Number 3”

### Quality control

After collection, specimens were stored in ethanol (96%) before sorting. Specimens, adults and juveniles, were identified in the laboratory by a trained parataxonomist (Sophie Wallon) and organised following a system of morphospecies (Oliver and Beattie 1996). Final identification was done by the senior author (Paulo A. V. B.).

For each species identified, a colonisation status (Endemic, Native (non-endemic), Introduced, Indeterminate) named as “establishmentMeans” in the Occurrence table, was attributed following Borges et al. (2022).

### Step description

Specimens were identified, based on the Azorean arthropods collection “Dalberto Teixeira Pombo Insect Collection (DTP), University of Azores” created and maintained by Professor Paulo A. V. B.. A new collection reference was created, in the framework of the project PASTURCLIM, referencing each species occurring in the present dataset. If the specimen observed did not correspond to species/morphospecies recorded in any specimen already recorded in the Azorean arthropods collection or if its identification was not possible, then a new morphospecies number was attributed to that specimen (identificationRemarks in Occurrence table).

## Geographic coverage

### Description

Terceira Island, Azores, Portugal.

### Coordinates

-27.394 and -27.0150 Latitude; 38.814 and 38.638 Longitude.

## Taxonomic coverage

### Description

The following classes and orders of arthropods are covered:

Arachnida: Araneae, Opiliones, Pseudoscorpiones; Chilopoda: Lithobiomorpha, Scutigeromorpha; Diplopoda: Julida, Polydesmida; and Insecta: Coleoptera, Dermaptera, Hemiptera, Hymenoptera, Lepidoptera, Neuroptera, Orthoptera, Psocoptera.

### Taxa included

**Table:** 

Rank	Scientific Name	Common Name
class	Arachnida	Arachnids
order	Araneae	Spiders
order	Opiliones	Harvestmen
order	Pseudoscorpiones	Pseudoscorpions
class	Chilopoda	Centipedes
order	Lithobiomorpha	Centipedes
order	Scutigeromorpha	Centipedes
class	Diplopoda	Millipedes
order	Julida	Millipedes
order	Polydesmida	Millipedes
class	Insecta	Insects
order	Coleoptera	Beetles
order	Dermaptera	Earwigs
order	Hemiptera	Bugs
order	Hymenoptera	Ants
order	Lepidoptera	Moths
order	Neuroptera	Lacewings
order	Orthoptera	Crickets, Grasshoppers
order	Psocodea	Psocids, Barklice, Booklice

## Temporal coverage

### Notes

Winter 2020 (03-2020):

Field A: 20 February 2020 till 5 March 2020 (14 days)

Field B: 26 February 2020 till 11 March 2020 (14 days)

Field C: 24 February 2020 till 9 March 2020 (14 days)

Summer 2020 (09-2020):

Field A: 24 August 2020 till 7 September 2020 (14 days)

Field B: 25 August 2020 till 7 September 2020 (13 days)

Field C: 27 August 2020 till 10 September 2020 (14 days)

## Collection data

### Collection name

Entomoteca Dalberto Teixeira Pombo at University of Azores

### Collection identifier

DTP

### Specimen preservation method

All specimens were preserved in 96% ethanol.

### Curatorial unit

Dalberto Teixeira Pombo insect collection at the University of the Azores (Curator: Paulo A. V. B.)

## Usage licence

### Usage licence

Creative Commons Public Domain Waiver (CC-Zero)

## Data resources

### Data package title

Monitoring grassland’s arthropods in an in situ climate change experimentation (Terceira, Azores, Portugal)

### Resource link

http://ipt.gbif.pt/ipt/resource?r=pasturclim_otc

### Alternative identifiers

https://www.gbif.org/dataset/2276a616-3da6-4528-89c4-bcefd34a4f6e

### Number of data sets

2

### Data set 1.

#### Data set name

Event Table

#### Data format

Darwin Core Archive

#### Character set

UTF-8

#### Download URL

http://ipt.gbif.pt/ipt/resource?r=pasturclim_otc

#### Data format version

Version 1.4

#### Description

The dataset is available on the Global Biodiversity Information Facility platform, GBIF (Wallon et al. 2023). The event table dataset is organied following the Darwin Core Archive (DwCA) format and contains 297 records (eventID).

**Data set 1.** 

Column label	Column description
eventID	An identifier for every single event and specific to the dataset.
samplingProtocol	The methods or protocols used during an Event.
sampleSizeValue	A numeric value for a measurement of the size (time duration, length, area or volume) of a sample in a sampling event.
sampleSizeUnit	The unit of measurement of the size (time duration, length, area or volume) of a sample in a sampling event.
samplingEffort	The amount of effort expended during an Event.
eventDate	Date or date range the record was collected.
year	Year of the event.
month	Month of the event.
verbatimEventDate	The verbatim original representation of the date and time information.
habitat	Description of the habitat in which the Event occurred.
fieldNotes	Note to facilitate the characterisation of the plot treatment: Control plot or plot surrounded by an Open Top Chamber.
locationID	An identifier for the set of location information (specific to the dataset).
islandGroup	Name of the archipelago of the sampling site.
island	Name of the island of the sampling site (Terceira Island).
country	Name of the country of the sampling site.
countryCode	The standard code for the country in which the Location occurs.
stateProvince	An identifier for every single event and specific to the dataset.
municipality	Municipality of the sampling site.
locality	Name of the locality.
minimumElevationInMetres	The lower limit of the range of elevation (altitude, usually above sea level), in metres.
maximumElevationInMetres	The highest limit of the range of elevation (altitude, usually above sea level), in metres.
decimalLatitude	Geographic coordinate (Decimal degrees): sampling location Latitude.
decimalLongitude	Geographic coordinate (Decimal degrees): sampling location Longitude.
geodeticDatum	Spatial reference system (SRS) upon which the geographic coordinates given in decimalLatitude and decimalLongitude are based.
coordinateUncertaintyInMetres	Coordinates' uncertainty in metres to the site of the true sampling area.
coordinatePrecision	A decimal representation of the precision of the coordinates given in the decimalLatitude and decimalLongitude.
georeferenceSources	A map, gazetteer or other resource used to georeference the Location.

### Data set 2.

#### Data set name

Occurrences table

#### Data format

Darwin Core Archive

#### Character set

UTF-8

#### Download URL

http://ipt.gbif.pt/ipt/resource?r=pasturclim_otc

#### Data format version

version 1.4

#### Description

The dataset is available on the Global Biodiversity Information Facility platform, GBIF (Wallon et al. 2023). The occurrence table dataset is organised following the Darwin Core Archive (DwCA) format and contains 6051 records (occurrenceID).

**Data set 2.** 

Column label	Column description
Event ID	An identifier for every single event and specific to the dataset.
type	The type of the related resource.
licence	Information about rights held in and over the resource.
institutionID	An identifier for the institution having custody of the object(s) or information referred to in the record.
collectionID	An identifier for the collection or dataset from which the record was derived.
institutionCode	The name in use by the institution having custody of the object(s) or information referred to in the record.
collectionCode	The acronym identifying the collection or dataset from which the record was derived.
datasetName	The name identifying the dataset from which the record was derived.
basisOfRecord	The specific nature of the data record.
occurrenceID	An identifier built as a "Globally Unique IDentifier".
recordedBy	Names of people responsible for recording the original occurrence.
organismQuantity	A number for the quantity of organisms.
organismQuantityType	The type of quantification system used for the quantity of organisms.
sex	The sex of the biological individual(s) represented in the occurrence.
lifeStage	The age class or life stage of the Organism(s) at the time the Occurrence was recorded.
establishmentMeans	The process of establishment of the species in the location, using a controlled vocabulary: 'native', 'introduced', 'indeterminate'.
occurrenceRemarks	Comments or notes about the Occurrence mentioning the 'endemic' species.
identifiedBy	Names of people who assigned the Taxon to the subject.
dateIdentified	The date on which the subject was determined as representing the Taxon.
identificationRemarks	Dalberto Teixeira Pombo (DTP) collection's morphospecies number attributed to specimens identified.
scientificName	Full scientific name, with authorship and date information, if known. When identification to species level was not possible, then it is the name in the lowest level taxonomic rank that can be determined.
kingdom	Scientific name of the kingdom in which the taxon is classified.
phylum	Scientific name of the phylum in which the taxon is classified.
class	Scientific name of the class in which the taxon is classified.
order	Scientific name of the order in which the taxon is classified.
family	Scientific name of the family in which the taxon is classified.
genus	Scientific name of the genus in which the taxon is classified.
subgenus	Scientific name of the sub genus in which the taxon is classified.
specificEpithet	The species epithet of the scientific name.
infraspecificEpithet	Name of the lowest or terminal infraspecific epithet of the scientific name.
taxonRank	The taxonomic rank of the most specific name in the scientific name.
scientificNameAuthorship	The authorship information related to the scientific name.

## Additional information

We collected a total 41,351 specimens belonging to four classes, 15 orders, 60 families and 171 morphospecies (including 34 taxa identified only at order, family or genus level). Therefore, 137 taxa have a scientific name associated (n = 38918) (from now on “species”) Table 2.

Regarding the colonisation status, introduced species (also those with an "indeterminate" colonisation status that are most probably exotic species (n = 7622)) represented 71% (n = 29664 specimens) of the total abundance and 75% (129 species) of the total richness; 28% (n = 11608 specimens) of the total abundance and 19% (33 species) of the total richness were represented by native non-endemic species; finally, endemic species represented 0.2% (n = 79 specimens) of the total abundance and 1% (one species) of the total richness.

Spiders (Arachnida, Araneae) and beetles (Insecta, Coleoptera) were the two most diversified and abundant groups.

Altogether, *Pseudoophonusrufipes* (De Geer, 1774) (Coleoptera, Carabidae), an omnivorous ground beetle, dominated the samples and represented 17% of the total arthropod abundance. This ground beetle dominated summer samples, while the predator rove beetle *Ocypusolens* (Müller, 1764) (Coleoptera, Staphylinidae) dominated winter samples.

The dominant spider was *Oedothoraxfuscus* (Blackwall, 1834) (Araneae, Linyphiidae) representing 5% of overall arthropod abundance. It was also the most dominant spider species in summer samples, while winter samples were dominated by the spider *Erigonedentipalpis* (Wider, 1834) (Araneae, Linyphiidae).

Some species distributions varied with elevation and consequently with the type of field. The ground-beetle *Notiophilusquadripunctatus* Dejean, 1826 (Coleoptera, Carabidae) dominated winter samples (n = 464, 14%) at the low altitude field (field A) and the European earwig *Forficulaauricularia* Linnaeus, 1758 (Dermaptera) was the most abundant arthropod in the summer samples (n = 3177, 24%) of the same field; at the intermediate altitude field (field B), the rove beetle *Ocypusolens* (Müller, 1764) (Coleoptera, Staphylinidae) (n = 579, 25%) dominated winter samples and the ground beetle *Pseudoophonusrufipes* (De Geer, 1774) (Coleoptera, Carabidae) (n = 5822, 61%) summer samples; finally, the rove beetle *Amischaanalis* (Gravenhorst, 1802) (Coleoptera, Staphylinidae) was the most abundant species during the winter (n = 211, 14%) in the upper altitude field (field C), while the harvestman *Leiobunumblackwalli* (Arachnida, Opiliones) (n = 3882, 33%) was the dominant species in summer.

Our study is responding to the need to have baseline data to understand long-term insect decline patterns (Seibold et al. 2019). Setting monitoring programmes using arthropods is important for understanding and managing pest populations, detecting environmental changes, assessing the impact of management practices and identifying potential threats to biodiversity (Borges et al. 2019).

## Figures and Tables

**Table 1.** Description of the locality, habitat, elevation and coordinates (in decimal degrees) of the three fields sampled in Terceira island, Azores.

Locality	Site Code	Habitat	Grass cover species	Elevation (m)	Longitude		Latitude
Santa Bárbara-Field_A	A	Pasture	*Loliummultiflorum*	186	-27.35381	38.70351	
Santa Bárbara-Field_B	B	Pasture	*Loliummultiflorum*	301	-27.32578	38.70164	
Granja da Universidade-Field_C	C	Pasture	*Holcuslanatus*	385	-27.17008	38.69777	

**Table 2.** Inventory of arthropods collected in three pastures (Fields A, B and C) in Terceira Island (Azores, Portugal) in control plots (C) and plots surrounded by an OTC (T). AC - Field A control plot; AT - Field A plot OTC; BC - Field B control plot; BT - Field B plot OTC; CC - Field C control plot; CT - Field C plot OTC. The list includes only the specimens identified at species-level. Class, order, family and scientific name follow alphabetical sequence. Colonisation statuses, based on Borges et al. (2022) and abundance per field and treatment, are provided. Colonisation status (Origin): END - Endemic; NAT - native non-endemic; INTR - introduced; IND - indeterminate.

**Family**	**Scientific Name**	**Origin**	**AC**	**AT**	**BC**	**BT**	**CC**	**T**	**Total**
**Arachnida**	**Araneae**								
Dysderidae	*Dysderacrocata* C. L. Koch, 1838	INTR	3	3	1	11	2	12	32
Gnaphosidae	*Marinarozeloteslyonneti* (Audouin, 1826)	INTR		1					1
Gnaphosidae	*Zelotesaeneus* (Simon, 1878)	INTR			2	2			4
Linyphiidae	*Agynetafuscipalpa* (C. L. Koch, 1836")	INTR	98	58		1	1	1	159
Linyphiidae	*Erigoneatra* Blackwall, 1833	INTR	3		4	1	182	67	257
Linyphiidae	*Erigoneautumnalis* Emerton, 1882	INTR	148	88	79	67	54	9	445
Linyphiidae	*Erigonedentipalpis* (Wider, 1834)	INTR	109	52	87	95	569	248	1160
Linyphiidae	*Mermessusbryantae* (Ivie & Barrows, 1935)	INTR	10	8	4	5	25	42	94
Linyphiidae	*Mermessusfradeorum* (Berland, 1932)	INTR	5	8	20	24	99	102	258
Linyphiidae	*Nerieneclathrata* (Sundevall, 1830)	INTR		1		1			2
Linyphiidae	*Oedothoraxfuscus* (Blackwall, 1834)	INTR	170	151	72	65	975	473	1906
Linyphiidae	*Osteariusmelanopygius* (O. Pickard-Cambridge, 1880)	INTR	23	43	12	44		3	125
Linyphiidae	*Prinerigonevagans* (Audouin, 1826)	INTR	13	9	2	5	13	4	46
Linyphiidae	*Tenuiphantestenuis* (Blackwall, 1852)	INTR	43	69	30	80	41	71	334
Lycosidae	*Pardosaacorensis* Simon, 1883	END				1	50	28	79
Mimetidae	*Erofurcata* (Villers, 1789)	INTR						1	1
Oecobiidae	*Oecobiusnavus* Blackwall, 1859	INTR	1						1
Tetragnathidae	*Pachygnathadegeeri* Sundevall, 1830	INTR			33	67	7	34	141
Theridiidae	*Cryptachaeablattea* (Urquhart, 1886)	INTR				3			3
Zodariidae	*Zodarionatlanticum* Pekár & Cardoso, 2005	INTR	3	3		1			7
**Arachnida**	**Opiliones**								
Leiobunidae	*Leiobunumblackwalli* Meade, 1861	NAT					2621	1313	3934
Sclerosomatidae	*Homalenotuscoriaceus* (Simon, 1879)	NAT	1	5	73	283	71	68	501
**Arachnida**	**Pseudoscorpiones**								
Chthoniidae	*Chthoniusischnocheles* (Hermann, 1804)	INTR		1					1
Neobisiidae	*Neobisiummaroccanum* Beier, 1930	INTR		1				1	2
**Chilopoda**	**Lithobiomorpha**								
Lithobiidae	*Lithobiuspilicornispilicornis* Newport, 1844	NAT	2	5	7	15	25	13	67
**Chilopoda**	**Scutigeromorpha**								
Scutigeridae	*Scutigeracoleoptrata* (Linnaeus, 1758)	INTR	13	39	1				53
**Diplopoda**	**Julida**								
Blaniulidae	*Blaniulusguttulatus* (Fabricius, 1798)	INTR	1					5	6
Blaniulidae	*Nopoiuluskochii* (Gervais, 1847)	INTR					2	1	3
Blaniulidae	*Proteroiulusfuscus* (Am Stein, 1857)	INTR	2	4				4	10
Julidae	*Cylindroiuluspropinquus* (Porat, 1870)	INTR	1	1	1		19	15	37
Julidae	*Ommatoiulusmoreleti* (Lucas, 1860)	INTR	504	278	21	25	60	186	1074
**Diplopoda**	**Polydesmida**								
Paradoxosomatidae	*Oxidusgracilis* (C.L. Koch, 1847)	INTR			2		7	10	19
Polydesmidae	*Polydesmuscoriaceus* Porat, 1870	INTR	107	72	108	164	215	276	942
**Insecta**	**Coleoptera**								
Anthicidae	*Hirticollisquadriguttatus* (Rossi, 1792)	NAT	3	2					5
Aphodiidae	*Calamosternusgranarius* (Linnaeus, 1767)	INTR	5	1		1			7
Apionidae	*Aspidapionradiolus* (Marsham, 1802)	NAT	1	13		1	6	11	32
Carabidae	*Agonummuellerimuelleri* (Herbst)	INTR			3				3
Carabidae	*Amaraaenea* (De Geer, 1774)	INTR			6		1		7
Carabidae	*Anisodactylusbinotatus* (Fabricius, 1787)	INTR	3	3	87	33	190	88	404
Carabidae	*Bembidionambiguum* Dejean, 1831	INTR	1	3	1	3	1		9
Carabidae	*Calosomaolivieri* Dejean, 1831	NAT	2	2	16	30	1	1	52
Carabidae	*Harpalusdistinguendusdistinguendus* (Duftschmidt, 1812)	INTR	139	198	23	13			373
Carabidae	*Laemostenuscomplanatus* (Dejean, 1828)	INTR	1	5					6
Carabidae	*Notiophilusquadripunctatus* Dejean, 1826	NAT	287	191	44	48			570
Carabidae	*Ophonusardosiacus* (Lutshnik, 1922)	INTR		1					1
Carabidae	*Paranchusalbipes* (Fabricius, 1796)	INTR					28	170	198
Carabidae	*Pseudoophonusrufipes* (De Geer, 1774)	INTR	247	127	3343	2480	285	466	6948
Carabidae	*Pterostichusvernalis* (Panzer, 1796)	INTR	3		19	6	567	709	1304
Carabidae	*Stenolophusteutonus* (Schrank, 1781)	NAT			1		30	5	36
Chrysomelidae	*Epitrixcucumeris* (Harris, 1851)	INTR	1						1
Chrysomelidae	*Epitrixhirtipennis* (Melsheimer, 1847)	INTR		1					1
Coccinellidae	*Rhyzobiuslophanthae* (Blaisdell, 1892)	INTR		1					1
Coccinellidae	*Scymnusinterruptus* (Goeze, 1777)	NAT	3			1			4
Coccinellidae	*Scymnusnubilus* Mulsant, 1850	NAT	3			1			4
Corylophidae	*Sericoderuslateralis* (Gyllenhal, 1827)	INTR	6	18		2	1	1	28
Curculionidae	*Coccotrypescarpophagus* (Hornung, 1842)	INTR		1	1			4	6
Curculionidae	*Mecinuspascuorum* (Gyllenhal, 1813)	INTR		1					1
Curculionidae	*Orthochaetesinsignis* (Aubé, 1863)	NAT		1					1
Curculionidae	*Sitonadiscoideus* Gyllenhal, 1834	INTR	49	16	8	24	10	2	109
Curculionidae	*Tychiuspicirostris* (Fabricius,1787)	INTR						2	2
Dryophthoridae	*Sitophilusoryzae* (Linnaeus, 1763)	INTR			1				1
Dryophthoridae	*Sphenophorusabbreviatus* (Fabricius, 1787)	INTR	31	10	8	7	5	3	64
Dryopidae	*Dryopsluridus* (Erichson, 1847)	NAT	1				14	11	26
Elateridae	*Aeolusmelliculusmoreleti* Tarnier, 1860	INTR	52	12	12	5	5		86
Elateridae	*Melanotusdichrous* (Erichson, 1841)	INTR	3	1			16	13	33
Hydrophilidae	*Cercyonhaemorrhoidalis* (Fabricius, 1775)	INTR	39	3	13	6	13	1	75
Hydrophilidae	*Sphaeridiumbipustulatum* Fabricius, 1781	INTR					1		1
Latridiidae	*Cartoderenodifer* (Westwood, 1839)	INTR						4	4
Mycetophagidae	*Litargusbalteatus* Le Conte, 1856	INTR	1						1
Mycetophagidae	*Typhaeastercorea* (Linnaeus, 1758)	INTR	15	11	3				29
Nitidulidae	*Carpophilusfumatus* Boheman, 1851	INTR					2	1	3
Nitidulidae	*Epuraeabiguttata* (Thunberg, 1784)	INTR	3				1		4
Nitidulidae	*Phenolialimbatatibialis* (Boheman, 1851)	INTR	1						1
Nitidulidae	*Stelidotageminata* (Say, 1825)	INTR	4	1	8	1	2		16
Phalacridae	*Stilbustestaceus* (Panzer, 1797)	NAT	1		1				2
Ptiliidae	*Ptenidiumpusillum* (Gyllenhal, 1808)	INTR	2	1	3	3	1	1	11
Scarabaeidae	*Onthophagustaurus* (Schreber, 1759)	INTR	1	1	11	13	5		31
Scarabaeidae	*Onthophagusvacca* (Linnaeus, 1767)	INTR		2	1	1			4
Staphylinidae	*Aleocharabipustulata* (Linnaeus, 1760)	IND	8	2	2		31	3	46
Staphylinidae	*Aleocharaverna* Say, 1833	IND					1		1
Staphylinidae	*Aloconotasulcifrons* (Stephens, 1832)	IND	13			1	6		20
Staphylinidae	*Amischaanalis* (Gravenhorst, 1802)	IND	36	21	39	68	408	110	682
Staphylinidae	*Amischaforcipata* Mulsant & Rey, 1873	IND	3				24	4	31
Staphylinidae	*Anotylusnitidifrons* (Wollaston, 1871)	IND	1429	621	125	33	284	47	2539
Staphylinidae	*Anotylusnitidulus* (Gravenhorst, 1802)	IND	9	3			3	3	18
Staphylinidae	*Astenuslyonessius* (Joy, 1908)	IND	1						1
Staphylinidae	*Athetaaeneicollis* (Sharp, 1869)	IND	1				3	12	16
Staphylinidae	*Athetafungi* (Gravenhorst, 1806)	IND	42	1					43
Staphylinidae	*Athetapalustris* (Kiesenwetter, 1844)	IND	2				2	5	9
Staphylinidae	*Athetapasadenae* Bernhauer, 1806	IND	3				5	3	11
Staphylinidae	*Carpelimuscorticinus* (Gravenhorst, 1806)	IND	1						1
Staphylinidae	*Carpelimuszealandicus* (Sharp, 1900)	INTR				6			6
Staphylinidae	*Coproporuspulchellus* (Erichson, 1839)	IND						1	1
Staphylinidae	*Cordaliaobscura* (Gravenhorst, 1802)	IND	265	61	80	50	152	75	683
Staphylinidae	*Gabriusnigritulus* (Gravenhorst, 1802)	IND	1				6	1	8
Staphylinidae	*Gyrohypnusfracticornis* (Müller, 1776)	IND	10	4	29	32	26	17	118
Staphylinidae	*Ocypusolens* (Müller, 1764)	IND	97	108	303	484	6	23	1021
Staphylinidae	*Oligotapumilio* Kiesenwetter, 1858	IND	18	57	2	16	12	24	129
Staphylinidae	*Oligotapusillima* (Gravenhorst, 1806)	IND	2	5		4			11
Staphylinidae	*Philonthuslongicornis* Stephens, 1832	IND					3		3
Staphylinidae	*Philonthusquisquiliariusquisquiliarius* (Gyllenhal, 1810)	IND						1	1
Staphylinidae	*Pseudoplectusperplexus* (Jacquelin du Val, 1854)	IND					2	1	3
Staphylinidae	*Quediussimplicifrons* Fairmaire, 1862	IND				2	13	14	29
Staphylinidae	*Rugilusorbiculatus* (Paykull, 1789)	IND	802	214	159	148	108	41	1472
Staphylinidae	*Sepedophiluslusitanicus* Hammond, 1973	IND	4	3			1		8
Staphylinidae	*Stenomastaxmadeirae* Assing, 2003	IND	12		1		54	11	78
Staphylinidae	*Suniuspropinquus* (Brisout de Barneville, 1867)	IND		3					3
Staphylinidae	*Tachyporuschrysomelinus* (Linnaeus, 1758)	IND	3					2	5
Staphylinidae	*Tachyporusnitidulus* (Fabricius, 1781)	IND	13	38	8	26	6	7	98
Staphylinidae	*Xantholinuslongiventris* Heer, 1839	IND	1	1	3	18	9	6	38
Tenebrionidae	*Blapslethifera* Marsham, 1802	INTR		1					1
**Insecta**	**Dermaptera**								
Anisolabididae	*Euborelliaannulipes* (Lucas, 1847)	INTR	1	5	20	185	2	20	233
Forficulidae	*Forficulaauricularia* Linnaeus, 1758	INTR	1802	1482	75	69	8	30	3466
**Insecta**	**Hemiptera**								
Anthocoridae	*Anthocorisnemoralis* (Fabricius, 1794)	NAT		2				1	3
Aphididae	*Rhopalosiphoninuslatysiphon* (Davidson, 1912)	INTR	4	17	1	1		1	24
Cicadellidae	*Anoscopusalbifrons* (Linnaeus, 1758)	NAT	24	15	16	45	22	6	128
Cicadellidae	*Euscelidiusvariegatus* (Kirschbaum, 1858)	NAT	1	3			4	2	10
Cydnidae	*Geotomuspunctulatus* (A. Costa, 1847)	NAT	245	60	22	6			333
Delphacidae	*Kelisiaribauti* Wagner, 1938	NAT			2	1	23	2	28
Delphacidae	*Megamelodesquadrimaculatus* (Signoret, 1865)	NAT					19	3	22
Lygaeidae	*Kleidocerysericae* (Horváth)	NAT			1				1
Nabidae	*Nabispseudoferusibericus* Remane, 1962	NAT	7	1	2	3	1		14
Rhyparochromidae	*Beosusmaritimus* (Scopoli, 1763)	NAT	18	4		1		1	24
Rhyparochromidae	*Scolopostethusdecoratus* (Hahn, 1833)	NAT	35	18	1	1			55
Saldidae	*Saldulapalustris* (Douglas)	NAT			1				1
**Insecta**	**Hymenoptera**								
Apidae	*Bombusruderatus* (Fabricius, 1775)	INTR				1			1
Formicidae	*Hypoponeraeduardi* (Forel, 1894)	NAT	89	91	308	161		2	651
Formicidae	*Lasiusgrandis* Forel, 1909	NAT	230	310	192	305	89	67	1193
Formicidae	*Linepithemahumile* (Mayr, 1868)	INTR	2	36	25	1		2	66
Formicidae	*Monomoriumcarbonarium* (F. Smith, 1858)	NAT	3	10					13
Formicidae	*Tetramoriumcaespitum* (Linnaeus, 1758)	NAT	1470	1296	204	89			3059
**Insecta**	**Lepidoptera**								
Noctuidae	*Mythimnaunipuncta* (Haworth, 1809)	NAT			5	1			6
**Insecta**	**Neuroptera**								
Chrysopidae	*Chrysoperlaagilis* Henry et al., 2003	NAT	1	1				7	9
Chrysopidae	*Chrysoperlalucasina* (Lacroix, 1912)	NAT	1	1				7	9
**Insecta**	**Orthoptera**								
Gryllidae	*Eumodicogryllusbordigalensis* (Latreille, 1804)	INTR	23	8	56	13	80	20	200
Gryllidae	*Gryllusbimaculatus* De Geer, 1773	INTR	9	9	5	2			25
**Insecta**	**Psocodea**								
Ectopsocidae	Ectopsocusbriggsi McLachlan, 1899	INTR						1	1
	**TOTAL**		**8909**	**6039**	**5859**	**5412**	**7636**	**5063**	**38918**
